# Family presence during resuscitation: adaptation and validation into Spanish of the Family Presence Risk-Benefit scale and the Self-Confidence scale instrument

**DOI:** 10.1186/s12913-021-06180-2

**Published:** 2021-03-12

**Authors:** Eva de Mingo-Fernández, Ángel Belzunegui-Eraso, María Jiménez-Herrera

**Affiliations:** 1 Universitat Rovira i Virgili, Departament d’Infermeria, Tarragona, Spain; 2 Consorci Sanitari de l’Alt Penedès i Garraf (CSAPG), Barcelona, Spain; 3 Universitat Rovira i Virgili, Fundació Martí-Franquès, Tarragona, Spain; 4 Universitat Rovira i Virgili, Departament d’estadística, Tarragona, Spain

**Keywords:** Family witnessed resuscitation, Ethic CPR, Family presence during resuscitation, Staff opinion, Family presence during resuscitation

## Abstract

**Background:**

Family Presence during Cardiopulmonary Resuscitation has been studied both to identify the opinions of health professionals, patients, and family members, and to identify benefits and barriers, as well as to design protocols for its implementation. R. Twibell and her team designed an instrument that measured nurses’ perceptions of Risks-Benefits and Self-Confidence regarding Family Presence during Resuscitation. There are few studies in Spain on this practice.

**Methods:**

The aim is to adapt and validate into Spanish the Family Presence Risk-Benefit scale and Family Presence Self-Confidence scale instrument. For this purpose, this instrument was translated cross-culturally, and administered in paper and online version. Statistical tests were carried out for the validity of the questionnaire. Five hundred forty-one healthcare professionals were invited to respond. The results were analyzed by the same statistical procedures as in the original scale. Ethical approvals and research permissions were obtained according to national standards.

**Results:**

Two hundred thirty-seven healthcare professionals (43.8%) answered the survey (69% women), of whom 167 were nurses. Validation of instruments: Cronbach’s α in Family Presence Risk-Benefit scale was 0.94. Cronbach’s α in Family Presence Self-Confidence scale was 0.96. Factor Analysis Kaiser, Meyer and Olkin (KMO) was greater than 0.9. The correlation between the two measured scales, is significant and has a moderate intensity of the relationship (r = 0.65 and α < 0.001).

A lower predisposition to Family Presence during Cardiopulmonary Resuscitation is observed, but the pure detractors are only 12%. Doctors are more reluctant than nurses.

**Conclusions:**

The psychometric properties of the questionnaire in Spanish indicate high validity and reliability. Risk-Benefit perception and Self-Confidence are related to the healthcare professionals who consider the Family Presence to be beneficial. More studies in different contexts are necessary to confirm the psychometric results and validity of this instrument in Spanish.

**Supplementary Information:**

The online version contains supplementary material available at 10.1186/s12913-021-06180-2.

## Background

The World Health Organization (WHO) concept of health allows for a holistic view of the human being, with attention focused on the person, by including the family and the communities in the center of the health system, since the individual develops within the family and the family becomes the first patient support system [1].

Family Presence During Resuscitation (FPDR) can be defined as the presence of one or more family members in the space where cardiopulmonary resuscitation (CPR) is being performed and that they are able to maintain visual or physical contact with the patient and healthcare team [2].

In 1982, at the Foote Hospital in Michigan in the United States, the protocols of the time, which did not allow FPDR, were challenged by the request of two families to be present during CPR, generating a debate about the right to be at the side of their relatives [3].

Family Presence during Resuscitation (FPDR) is controversial. From the health professionals’ point of view, some authors point out, as general arguments in favor, that it offers the opportunity to give emotional support to the patient and to be by the side of the loved one, reducing at the same time the anxiety of the relatives. It also allows to follow the events at all times, which facilitates making quick and consensual decisions in situ [4, 5], and enabling a better process of grief [6].

The arguments to the contrary focus on several aspects, such as that the medical team feels evaluated and analyzed, thus causing them discomfort and pressure [5, 7–9]; the frequency with which lack of knowledge and misinterpretation of procedures by family members give rise to traumas, complaints and fears of a possible interruption of proceedings; and also the limited physical space and legal vacuum, since there are no guidelines in this regard [8, 10, 11]. Relatives who have witnessed CPR think that it is beneficial for the patient and families, and this opinion is not conditioned by age, education or income, nor by the last will and testament [12]. Other determining factors for the acceptance of FPDR are of cultural and religious nature, varying the percentages of acceptance of FPDR when the studies come from countries with different cultural and religious traditions [13–19].

The European Resuscitation Council (ERC), a component of the International Liaison Committee on Resuscitation (ILCOR), in its 2015 ethical recommendations, states that the medical team should offer selected family members the opportunity to be present during the resuscitation of a loved one, the health team should be sensitive to FPDR, assigning a member of the team (if possible) to inform, support and give comfort to the family member present [20]. Additionally, the European Federation of Critical Care Nursing associations (EfCCNa) and the European Nursing Organizations support the FPDR [21, 22].

From an ethical point of view, hospital policies restricting FPDR should be rectified, and new protocols should be developed to enable admitting the family to these procedures, as the principle of patient’s autonomy supports this.

The primary aim of our study is to adapt and validate the scale of Twibell et al., since we found that we were not able to use it in our language. This study is necessary to obtain a reliable tool in Spanish, which would facilitate further studies on FPDR. The secondary aim was to know the opinions of health professionals (both medical and nursing staff) who work in different health services of the Consorci Sanitari del Garraf (CSG) of Barcelona, Spain, such as the Intensive Care Unit (ICU), Emergencies (ER), Dialysis, Social Healthcare (SH), Hospitalization facilities, etc., and thus objectify the opinions and preferences of the doctors and nurses carrying out the pre- and intrahospital services. To this end, the three scales from the study of Twibell et al. [23] are replicated: Family Presence Risk-Benefit (FPRB), Family Presence Self-Confidence (FPSC) [23] and also, a sociodemographic questionnaire. These indices measure the perceptions of doctors and nurses about the risks-benefits and self-confidence that the presence of the family entails in CPR. The transcultural translation of the instrument was performed, including the physicians in it, and the results obtained in the original study were compared with those obtained in the Garraf region (Barcelona). There are few studies in Spain on this practice and we translated this instrument so that further studies on FPDR can be carried out.

## Methods

Ethical approvals and research permissions were obtained according to national standards, guaranteeing the anonymity of the respondents. The study was approved by the institutional review board of the Consorci Sanitari del Garraf, and by the clinical research Ethic Committee of the Fundació Unió Catalana Hospitals with code CEIC 15/31.

The questionnaires elaborated by Twibell et al. [23] on FPRB-FPSC were selected because they provide high level of internal consistency and construct validity, following maximum likelihood exploratory factor analysis with varimax rotation. These scales are a valuable method to evaluate professionals’ perceptions of the risks, benefits, and self-confidence to manage FPDR. They consist of 3 questionnaires, a socio-demographic one, another one that evaluates Risk-Benefit (FPRB) and the last one that evaluates Self-Confidence (FPSC). The FPRB scale requires participants to rate their agreement with 22 items using a five-point Likert scale which ranges from strongly disagree (1) to strongly agree (5). A higher score indicates a greater level of the benefit perceived from FPDR. Only one factor was identified, accounting for 53% of variance in nurses’ perceptions of risks and benefits of FP. Factor loadings ranged from 0.890–0.0498 and internal consistency was Cronbach’s alfa of 0.96.

The FPSC scale also requires participants to rate their agreement with 17 questions using a five-point Likert scale, which ranges from not at all confident (1) to very confident (5). A higher score indicates a greater level of self-confidence in managing FPDR. Also, only one factor was identified for the 17 items, accounting for 52% of respondents’ perceptions. The FPSC factor loadings ranged from 0.553–0.825 and internal consistency was Cronbach’s alpha of 0.95.

Some items of the sociodemographic variables have been changed (Table 1) to fit the ethnic, professional, and educational profile of our country. Doctors were included to obtain a more generalized view of the subject. Initially, the questionnaire was applied to nurses, but other studies such as those of MC Lean [24] and Chapman [25] included doctors in them, which allowed to make comparisons between the two health groups.

**Table 1:** Table of amendment questions Questionnaire in Spanish

Twibell et al. [23] Original tool.			CSG (2018) Modifications.
**47.**	**In what unit were you working the last time when you invited a family member to be present during a resuscitation attempt?**	**47.**	**In what unit were you working the last time when you invited a family member to be present during a resuscitation attempt?**
	____Emergency Department		1) Emergencies 2) ICU 3) Social Healthcare
	____Critical Care Unit		4) Acute hospital unit 5) Residency
	____Non-Critical Care Inpatient Unit ____ Other		6) External Consultations 7) Hospital/day center
	____ Not applicable.		8) Rehabilitation 9) Others__________
**51.**	**What type of unit do you work on most often?**	**51.**	**In which unit do you work most often?**
	____Emergency Department		1) Emergency D. 2) ICU 3) Social Healthcare
	____Critical Care Unit		4) Acute hospital unit 5) Residency
	____Non-Critical Care Inpatient Unit ____ Outpatient Unit		6) External Consultations 7) Hospital/day center
	____Other		8) Rehabilitation 9) Others__________
**52.**	**Current nursing role**	**52.**	**Profession:**
	____RN		1.- Nurse _______
	____LPN		2.- Physician _______ 3.- Others_____
**54.**	**Highest nursing degree completed**	**54.**	**Highest level of education completed:**
	____Licensed Practical Nurse Program		1.) Graduate/Qualified
	____Associate Degree in Nursing		2.) Master’s Degree
	____Baccalaureate Degree in Nursing		3.) Doctorate
	____Master’s Degree in Nursing		
	____Doctoral Degree in Nursing		
**56.**	**Age**	**56.**	**Age**
	____18–24 years		1) 20–25 years 2)26–35 years
	____25–39 years		3) 36–45 years 4) 46–55 years
	____40–55 years		5) Over 55 years
	____Over 56 years		
**58.**	**Do you hold membership in a professional nursing organization?**		removed
	____Yes		
	____No		
**59.**	**Ethnicity**		removed
	____African-American ___ Asian		
	____Caucasian ___ Hispanic		
	____Native American ___ Eskimo		
	____Pacific-Islander ___ Other________		

The inclusion criteria was to be a doctor or a nurse working in the CSG. A convenience sample was created. It was formed of 541 CSG health professionals, of whom 197 are doctors and 344 nurses. They were invited to respond anonymously to this survey. The invitation to participate in the survey was sent by the main researcher, via corporate mail in a direct message with a link to Google Forms, from September 1st, 2018 to October 31st, 2018 and from May 1st, 2019 to June 30th, 2019. The respondents were given the option to answer to the survey on paper or online, via Google forms. To collect the paper surveys, cardboard boxes were provided, notifying the day of collection so that the anonymity of the respondents could be respected. To calculate the margin of error, a 95% confidence level was selected, with simple random sampling.

### Transcultural translation and validation of the instrument

The survey by Twibell et al. [23] was selected for transcultural translation. The methodology used for the cross-cultural adaptation of questionnaires for use in health research was the one proposed by Sousa and Rojjanasrirat [26]. Two native Spanish translators with fluent English translated it into Spanish and two native English translators translated it back into English so that the questions were similar and had the same meaning as in the original survey. In an initial pilot study, 25 surveys were completed to determine if the healthcare professionals understood the questions; comprehension problems arose with 3 of them. A Delphi group was convened with research, resuscitation, and ethics experts, and the 3 questions were resolved so that the final questionnaire could be developed.

To validate that the items measured the same underlying dimension, factorial analysis was performed using the maximum likelihood method, as in Twibell et al. [23], with all of the variables and not excluding them from the analysis due to the existence of low item-total correlations.

Next, the validity of the indices of the instrument caused by transcultural translation was questioned, calculating its reliability measures (item-total correlation) and internal consistency (Cronbach’s alpha). Correlations were performed between the Risk-Benefit and Self-Confidence indices, using Pearson’s r. Analysis of variance (ANOVA) was also used to relate the indices of the scales with attitudes, actions and characteristics of the sample when there were more than two groups, and Student’s t-test was used to compare means between two groups (according to independent variables).

### Comparison with the original survey and with other studies that have used the same instrument

After obtaining the statistical results, they were compared with the results of the other studies that have used the instrument of Twibell et al. [23]. In this comparison, the same statistical procedures were used as in the original survey. Then, a comparison was made with the data resulting from the combination of the sociodemographic scales and the FPRB and FPSC from several studies that used the same instrument.

## Results

The sample is composed by 237 health professionals, who represent 43.8% of the hospital’s professional population, and with a confidence level of 95%, the sample margin of error was 4.78%.

The survey was distributed among doctors and nurses. Regarding the total population of doctors and nurses, 47.4% of the doctors and 36.6% of the nurses of the hospital participated in the study. There is a higher presence of women (69%) than men (31%). In the sample, 48% of the health professionals are between 36 and 55 years of age, and 17% are older than 55 years. Fifty-one percent of the sample is composed of health professionals with more than 16 years of experience, with 24% of healthcare professionals having less than 5 years of experience and 51% of the sample having some specialty in their profession (Table 2).

**Table 2:** Descriptive results

QUESTIONS	Variables	% CSG Staff	QUESTIONS	Variables	% CSG Staff
55.- Gender	Man	31%	57R.- Do you hold a specialty in your profession?	No	49%
	Woman	69%		Yes	51%
56.- Age (years)	20–25	10%	48.- Who should make the decision about family presence during resuscitation efforts?	Physician	67%
	26–35	25%		Patient	46%
	36–45	24%		Nurse	42%
	46–55	24%		Family	33%
	Over 55	17%		Others	11%
54.- Highest level of education completed	Graduated/Army Professional	48%	46.-How many times have you invited a family member to be present during a resuscitation?	> 5 times	4%
	Masters	46%		< 5 times	5%
	Doctorate	6%		Never	92%
53.- Years of professional experience	Less than one year	5%	44.-Would you want your family members to be present if you are a patient who needs resuscitation?	No	73%
	1–5	18%		Yes	27%
	6–10	14%	45.- Have you ever been present in the room during the resuscitation of one of your family members?	No	87%
	11–15	11%		Yes	13%
	16–20	17%			
	Over 20 years	34%	47.- In what unit were you working the last time that you invited a family member to be present during a resuscitation attempt?	Emergencies	16%
51.- In which unit do you work most often?	Acute Hosp. Unit	27%		Acute Hosp. Unit	14%
	Emergencies	25%		Social healthcare	9%
	Social healthcare	22%		ICU	5%
	ICU	6%		External Consultation	1%
	External Consultation	5%		Residency	1%
	Residency	3%		Others	53%
	Hospital/Day center	1%	52.- Profession	Nurse	69%
	Rehabilitation	0%		Physician	30%
	Others	11%		Others	1%

It is relevant that the sample is made up mostly of health professionals who work in hospital, emergency, or social health care settings, so that it can be inferred that the information provided is based on their own experiences, since the interviewees work in areas where resuscitation is performed on a regular or occasional basis. In the survey, some questions take the FP issue to the personal sphere of the participants, and most of their responses were in the direction that they would not like their family members to be present during their resuscitation, especially for health professionals who work in the emergency room. Those who opted for their family members to be present are those who perceive more benefits in the FPDR. Healthcare providers who have experienced CPR barely reach 13%, with the majority being men and older people. As far as the invitation to the FPDR is concerned, it barely reaches 5% and is recurrently 4% (Table 3).

**Table 3:** Value Chisq and Cohen’s d

QUESTIONS	44	45	46	49a	49b	50	QUESTIONS	
**AGE (%)**	27,4%	12,7%	8,4%	45,6%	26,2%	65,8%	44	If I were a patient under resuscitation, I would like my family to be present in the room.
20–35 (*n* = 83)	25%	6%	7%	31%	39%	72%		
36–55 (*n* = 113)	28%	15%	9%	47%	24%	66%		
> 55 (*n* = 41)	29%	20%	10%	71%	7%	51%		
Value chisq	0,3	5,63	0,27	17,34	14,44	5,44		
*P*-value chisq	0,86	0,06	0,87	0,00	0,00	0,07		
Cohen’s d	–	–	–	0,56	0,51	–		
**GENDER**							45	I have been present in the room during the resuscitation of one of my relatives.
MEN (*n* = 73)	30%	22%	11%	49%	29%	60%		
WOMEN (*n* = 164)	26%	9%	7%	44%	25%	68%		
Value chisq	0,39	8,18	0,87	0,6	0,37	1,44		
*P*-value chisq	0,53	0,00	0,35	0,44	0,54	0,23		
Cohen’s d	–	0,38	–	–	–	–	46	I have sometimes invited a family member to be present during resuscitation.
**PROFESSIONAL STATUS**								
Nurse (*n* = 163)	31%	12%	9%	38%	31%	72%		
Doctor (*n* = 72)	21%	14%	7%	63%	14%	51%		
Value chisq	2,42	0,12	0,33	12,05	8,34	9,81		
*P*-value chisq	0,12	0,73	0,57	0,00	0,00	0,00		
Cohen’s d	–	–	–	0,46	0,38	0,41	49a	Believe the doctor is the most adequate person to decide about FPDR
**HOSPITAL UNIT**								
Acute Hosp Unit (*n* = 63)	35%	16%	11%	48%	24%	75%		
Emergency (*n* = 59)	14%	14%	3%	46%	27%	56%		
Social Healthcare (*n* = 51)	39%	4%	8%	27%	37%	80%	49b	Believe THE PATIENT is the most adequate person to decide about family presence during resuscitation
Value chisq	15,45	6,74	5,68	9,06	5,15	17,34		
*P*-value chisq	0,00	0,08	0,13	0,03	0,16	0,00		
Cohen’s d	0,53	–	–	0,4	–	0,56		
**YEARS OF EXPERIENCE**								
< 5 (*n* = 56)	25%	5%	0%	30%	43%	64%	50	The decision about family presence must be part of a patient-authorized advance directive
6–20 (*n* = 100)	33%	13%	9%	45%	31%	78%		
> 20 (*n* = 81)	22%	17%	14%	57%	9%	52%		
Value chisq	2,83	4,28	7,97	9,35	22,16	13,68		
*p*-value chisq	0,24	0,12	0,02	0,01	0,00	0,00		
Cohen’s d	–	–	0,37	0,41	0,64	0,5		

Regarding the question of who should decide on the FPDR, 67% reported that it was the doctor, and 46% that the patient; 42% believe that it should be the nurse and 33% the family, but if they were made to choose the most appropriate person to make this decision, the majority (46%) appointed the doctor, and only 26% believed that it should be the patient.

It is the doctors themselves who believe that they should make this decision, as well as the health professionals with more experience and age, and those who believe that there are more risks related to the presence of family members, and also those who feel less confident in the presence of family members during resuscitation (Table 3).

Health care professionals who work in the social health field, the youngest and least experienced respondents, and those who have higher self-confidence in the presence of family members believe that it is the patient who should decide.

Regarding the willingness to include FP in advance directives, 66% of healthcare professionals are in favor.

Emergency medical and nursing staff see more risks and fewer benefits to FPDR and rely less on themselves to face this possibility. On the other hand, hospital and social health workers see more benefits than risks, with those in the social health field being more self-confident.

A total of 76% of participants in the survey responded the open question about the main reasons for not offering the FPDR, and there were three reasons: emotional impact (42%), fear of the families’ disruptive reaction (25%) and distrust of the resuscitation team not feeling comfortable and unable to work under pressure (22%), all of them conditioning the results of the procedures. The three main reasons in favor of offering FPDR are: the request of the family members themselves (21%) (by the patient or family), the fact that families can verify that everything possible has been done for their loved one (20%) and can better understand the resuscitation procedure, and the accompaniment between the family and the patient during CPR (17%).

In the survey questions from 44 to 50 (see Table 3), participants are directly asked about their personal opinion and experiences of Family Presence. Most of responses in this set of questions were in the direction that they would not like their family members to be present if they were resuscitated. Specifically, the most reluctant healthcare professionals are those who work in the emergency care.

The prevailing view is that it is the physician who should make the decision on the FPDR and that the patients should include it in their advance directives.

The correlation between the two scales (RB/SC) is significant and with a moderate intensity of the relationship (r = 0.65 and α < 0.001).

To measure the effect size, Cohen’s d was incorporated, following the instructions of J. Cohen [27].

### Validity of the instrument

To validate the instrument, its reliability indices were calculated: Cronbach’s alpha and item-total correlations. In the Risk-Benefit index, there were five items [4, 8, 12–14] identified as poorly correlating with the total (r < 0.3), so they were removed. After the removal of these items, the calculations of the Risk-Benefit index yielded a Cronbach’s alpha of 0.95 (Table 4).

**Table 4:** Results CSG health personnel FPRB-FPSC

Perceptions of Self-Confidence in FPDR.		Loads	Loads	Loads	Risk-Benefit Perception in FPDR		Loads	Loads	Loads
Item number (original order)		F1	F2	F3	Item number (original order)		F1	F2	F3
		34%	20%	11%			24%	21%	14%
1	I could inform the relatives present during the resuscitation procedure.	0.666			1	Family members should be given the option of being present while their loved one is undergoing an invasive procedure.	0.761		
2	I could administer medication during a resuscitation witnessed by relatives		0.882		2	Family members would be terrified to witness the technique of an invasive procedure.	−0.477		
3	I could administer electrical therapies (defibrillation, cardioversion, etc.) during family-witnessed resuscitation.		0.904		3	Family members will have long-term difficulties in coping with the emotional impact of having witnessed a resuscitation.	−0.54		
4	I could perform thoracic compressions during resuscitation with relatives present.		0.866		4	The resuscitation team could develop a close relationship with family members who witness resuscitation efforts compared to family members who do not witness them.	0.551		
5	I could communicate effectively with the rest of the healthcare team during resuscitation witnessed by family members.	0.52	0.57		6	I would like to be present if a loved one were revived.	0.54		
6	I could maintain the dignity of the patient during a family-witnessed resuscitation.	0.591			7	Patients DO NOT want their relatives to be present during resuscitation attempts.	−0.522		
7	I could identify the relatives that have adequate behaviors during resuscitation.	0.611			9	Family members who witness a failed resuscitation will have a better and healthier grieving process.	0.546		
8	I could prepare the family to access the area of resuscitation of their loved one.	0.76			10	If my loved one is being resuscitated, they should allow me to be present since I am a nurse/ doctor.	0.365		
9	I could ensure that the doctors who assist the patient support the family presence during the resuscitation of their loved one.	0.572			11	The presence of family members will interfere with the resuscitation process.	−0.632		
10	I could accompany the relatives who are in the area of resuscitation during the resuscitation of their loved one.	0.777			15	Family members in the unit where I work would like to be present during resuscitation.			
11	I could inform the healthcare team that resuscitation is being witnessed by relatives.	0.542			16	The FPDR is beneficial for patients.	0.418		
12	I could provide comfort measures to the family members present during the resuscitation of their loved one	0.818			17	The FPDR is beneficial for families.	0.504		
13	I could identify the spiritual and emotional needs of the family members present during the resuscitation of their loved one.	0.777			18	The FPDR is beneficial for nurses.			0,796
14	I could encourage family members to talk with their loved one during resuscitation.	0.688			19	The FPDR is beneficial for physicians.			0,877
15	I could delegate functions to other nurses to support the family members present during the resuscitation of their loved one.	0.621			20	The FPDR should be part of family-centered care	0.564		
16	I could inform the family after resuscitation.			0.952	21	The FPDR will have a positive effect on the patient satisfaction survey of hospital care.		0,802	
17	I could coordinate the follow-up of the grief process of the relatives after a resuscitation if necessary.	0.517			22	The FPDR will have a positive effect on the survey of hospital satisfaction by the family.		0,811	
					23	The FPDR will have a positive effect on the nursing care satisfaction survey of the nurse.		0,896	
					24	The FPDR will have a positive effect on the physician hospital care satisfaction survey		0,892	
**Varimax rotation with 5 removed items in FPDR in Risk-Benefit**					25	The presence of family members during resuscitation is a right that all patients should have.	0,652		
**Maximum Likelihood method**					26	The presence of family members during resuscitation is a right that all family members should have.	0,618		

In the Self-Confidence index, the correlation of all items with the total was greater than 0.5, so no changes were necessary. Cronbach’s alpha in this construct equals 0.94 (Table 4).

Applying factor analysis, the KMO measure is greater than 0.9, which indicates the suitability of the variables. The Risk- Benefit and Self-confidence indices maintain a moderate correlation of r = 0.65 in a positive sense.

In the analytical sense of the Index of Risks and Benefits of the presence of relatives in our replica, three realities are measured:

- Factor 1: the role of patients and relatives in resuscitation (rights, attitudes, effects of FP on them...)
- Factor 2: benefits of FP for doctors and nurses.
- Factor 3: effects of FP on satisfaction surveys

In the Self-confidence Index: we find TWO realities, two different expressions of the concept of self-confidence:

- Factor 1: self-confidence towards the relationship with the family during resuscitation
- Factor 2: self-confidence towards the technical aspects of professional practice during resuscitation (administration of medication, electrical therapy, chest compression...)

## Discussion

Compared with the study by Twibell et al. [23] and in reference to the validity of the indices, a more dispersed distribution has been obtained, with higher indices of asymmetry and kurtosis, to the point that the self-confidence index does not comply with the principle of normality. In both questionnaires, the Cronbach’s α is very similar, with our FPRB being α = 0.95 in RB and α = 0.96 that of Twibell et al. [23]; FPSC obtained an α = 0,94 while Twibell et al. [23] obtained α = 0.95, so we can affirm that we were able to replicate the reliability of both indices. As for the correlation between the two scales (FPRB-FPSC), it is significant and has a moderate intensity of the relationship (r = 0.65 and α < 0.001), while the one by Twibell et al. [23], gave an r = 0.56 and an α < 0.001, slightly below the results from this study.

In the factorial analysis, six variables have been found not to work well in the Risk-Benefit index, three of them have been removed from both studies and the remaining ones have either been excluded or obtained low factorial loads in one or the other study (Table 4).

The fundamental difference with Twibell et al. [23] is that in our study, there are additional variables that have generated different correlations in certain factors. Thus, it is worth asking what underlying dimensions they were meant to measure: risks, benefits, or self-confidence, since different meanings of these concepts emerge from the results, either due to polysemy or because they are items that do not define these dimensions well. For example, polysemy was found in the self-confidence index, since health care providers’ self-confidence behaves differently if one speaks from the social perspective (treatment of families, communication), in that those who agree on FP are more confident and those who disagree are less confident, or if one speaks of technical procedures that are assumed to be performed whether they agree or disagree with FP.

The perceptions of the effect of FP on satisfaction surveys measure a risk or a benefit, since in all combinations of factor analysis, these are variables that do not correlate with the rest of the index items.

It does not seem that transcultural translation has produced any disturbance, but rather that the healthcare professionals participating in this study have different opinions than health professionals in the United States sample.

For the comparison of the sample, the inclusion of doctors in the study has made it possible to increase the presence of men and older professionals with more extensive experience, although the differences between medical and nursing staff have not been significant in most of the variables, as in the McLean [24] and Chapman [25] studies, which did include physicians in the sample. The sample in this study, although smaller (*n* = 237), is more representative in terms of hospital units (Table 5).

**Table 5:** Comparative sociodemographic data

RESULTS TWIBELL et al. [23]		%	RESULTS CSG HEALTH PROFESSIONALS		%
GENDER	Woman	95.7	GENDER	Woman	69.2
	Man	2.1		Man	31.8
AGE (years)	Less than 24 years	4.5	AGE (years)	From 20 to 35	35.0
	From 25 to 39	38.1		From 36 to 55	47.7
	From 40 to 55	46.1		More than 55	17.3
	More than 55	8.5	Years of experience	Less than 1	5.5
Years of experience	Less than 1	3.7		From 1 to 5	18.1
	From 1 to 5	18.4		From 6 to 10	14.3
	From 6 to 10	21.9		From 11 to 20	27.8
	From 11 to 20	30.7		More than 20 years	34.2
	More than 20 years	23.5	Highest level of education completed	Graduate	48.1
Highest level of education completed	Graduate	78		MA	
	MA			Assimilated/Qualified	
	Assimilated/Qualified			Postgraduate/Doctorate	51.9
	Postgraduate/Doctorate	3.7			
	Auxiliary nurse				

We observe that in comparison with the sample of Twibell et al. [23], in this study we have obtained a much smaller presence of professionals who have sometimes invited a family member to witness resuscitation, which makes it impossible to evaluate this item, since there were barely 20 cases.

A lower predisposition to FPDR is observed, due to a higher risk perception and lower self-confidence than in Twibell et al. [23], and this is the main result obtained from this study. This can be explained taking into account the different characteristics of the sample, especially the participation of doctors, who are the ones that value PF the worst, but we believe that the key is in the cultural differences, since in this study there are a lot fewer professionals that have ever invited the relatives to attend a resuscitation process and this constitutes a conditioning pattern that can influence all the rest. A difference has been observed between the assessments of the emergency staff, which would be a very interesting subject for further investigation.

An index correlation very similar to that of Twibell et al. [23] has been obtained: the medical and nursing professionals who perceive more benefits are the ones who are more confident of being able to manage the presence of the families.

Another key difference is that in this study no single explanatory factor has been obtained to explain the perception about inviting or not inviting relatives to resuscitation, and that the qualitative questions have allowed to confirm that the main barriers to invite relatives coincide with the theoretical framework presented by Twibell et al. [23]: avoidance of causing an unpleasant impact on families, fear of a disruptive reaction from families, and fear of the resuscitation team not working comfortably. However, the main reasons for inviting family members presented by Twibell et al. [23] in their study are not the ones most frequently mentioned by the participants of our study. The fact that families understand that everything was done for their loved one is one of the reasons mentioned by one in five participants, positive grief management is mentioned by one in ten, not emphasizing the understanding of the severity of the patient.

Overall, the FPDR rating is more positive than negative. In relative terms to the index averages, we have 48% of “detractors” but in absolute terms only 12% of the participants are pure detractors, because the scores of both indexes are below 2.5.

Doctors are more reluctant to FPDR than nurses, especially in emergency care and intensive care units. This aspect contrasts with the McLean [24] and Chapman [25] studies, where there are no significant differences in the perceptions of self-confidence, but it must be pointed out that in the center where the McLean study was carried out, training programs and protocols on PFDR were implemented, thus conditioning this perception. Also, professionals with specialties were found to be more reluctant, but this variable is very much influenced by the profession, since it is doctors who have specialties and most nurses do not have them, in part due to the recent incorporation of specialties in Spain. It is interesting to note that it is in the area of emergency care where they are most reluctant to FPDR, since it is the area where they have contact with this procedure most often, and also that it is in the social health field, where these procedures are hardly performed, where they have the most self-confidence, a result that could open doors to future investigations.

When asked who should make the decision, those who choose to give more responsibility to medical staff are those who see more risk in FP and who have less confidence in themselves (especially older, more experienced physicians). In contrast, those who see greater benefits and have more self-confidence (nurses and young professionals with less than 5 years of experience), choose to give the decision responsibility to the patient.

It is observed that, behind the attitudes, there is a background of generational change that is advancing to allow FP. In this regard, the same trend is observed when we ask whether FP should be part of the patient’s advance directives.

In our study, many professionals (26%) report a lack of specific training and protocols to successfully perform FPDR, a factor that coincides with the studies by Chapman [25] and Powers [28] stating that “a key predictor to support FPDR is to receive training and have specific protocols”.

It would be interesting to reproduce this tool in other Spanish-speaking contexts, in order to analyze its reliability outside the context studied.

### Limitations

The results of this study should be interpreted with caution due to their limitations. One limitation was related to the method of data collection (manual and electronic). Almost 50% of the population participated in the study. The relatively small sample can possibly be explained by the fact that this topic is still quite unknown and may cause uncomfortable emotions, which could negatively impact the willingness to participate in the study.

While in Twibell et al. [23], the original study obtained data that did not include a “do not know/no answer” option for each question, this study provided data that did take this answer option into account. There are advantages to this decision, since it does not force the participants to choose an answer when faced with doubts, but on the other hand, it can sometimes be used as an evasive category when faced with a decision in a specific situation.

Medical and nursing staff, who have at some point witnessed a loved one’s CPR, are mostly men and there may be a gender bias in FPDR.

A greater perception of risk and lower self-confidence is observed, especially among emergency care providers, than in the sample by Twibell et al. [23]. Health professionals with greater self-confidence consider FP to be more beneficial.

The difference of 12 years between the original study and this study must be taken into account, since society’s opinion and the training of health professionals change with regard to the principles of bioethics, especially that of autonomy, which gives more empowerment to the patient and the family, leaving behind clinical decisions of medical paternalism.

## Conclusions

The proposed indices are valid with the variables with which they are constructed since they offer high reliability indices. However, there are some variables that have been removed due to low correlation, as in the study by Twibell et al. [23]. Some of the variables have generated additional unexpected factors. Of the 26 variables of the risk index, 14 variables that measure the same thing have been secured. Out of the 17 variables in the self-confidence index, 13 are part of the same factor. It is worth reflecting on whether it is preferable to discard some of the variables that now form part of the indexes or whether the fact that the index has different dimensions constitutes a value to be preserved.

FP generates controversy among health professionals, with doctors and nurses differing in their opinions, the latter being more in favor of FP. There is a trend towards generational change, as younger and less experienced health professionals tend to have greater acceptance of FP.

A possible explanation for the different attitudes toward FPDR among health professionals in different countries could be related to cultural differences and different healthcare models.

More studies on FPDR are needed in Spanish-speaking regions, and therefore it would be advisable to replicate this study to ensure the reliability and validity of the instrument in different health and sociocultural contexts.

## Supplementary Information

**Additional file 1.**

## Data Availability

The data sets used and/or analyzed during the current study are available from the corresponding author upon reasonable request.

## Abbreviations

CPR: Cardiopulmonary Resuscitation
CSAPG: Consorci Sanitari de l’Alt Penedés i Garraf
CSG: Consorci Sanitari del Garraf
FP: Family Presence
FPDR: Family Presence during Resuscitation
FPRB: Family Presence Risk Benefit scale
FPSC: Family Presence Self Confidence scale
FWR: Family Witnessed Resuscitation
WHO: World Health Organization
