# Supplementary material for: Family presence during resuscitation: adaptation and validation into Spanish of the Family Presence Risk-Benefit scale and the Self-Confidence scale instrument
Source: BMC Health Serv Res. 2021 Mar 12;21:221. doi: 10.1186/s12913-021-06180-2 (PMC7953805; doi:10.1186/s12913-021-06180-2)
Supplement: Supplementary file 1 — Additional file 1. [file 12913_2021_6180_MOESM1_ESM.doc]

# Nurses’ Perceptions of Family-Witnessed Resuscitation

Across the nation, health care professionals, patients and families are debating the issue of having family members present when a loved one is being resuscitated. As an RN or LPN at BMH, your opinions about this matter are of interest to us. Completing this questionnaire is **voluntary. Please do not put your name on the survey.**

Definition: Family-witnessed resuscitation means one or more family members are present in the room while a loved one is being resuscitated in an effort to sustain life.

| **Please circle the number that best represents your opinion.** | | | Strongly Disagree | | Disagree | | Neutral | | Agree | Strongly Agree |
| --- | --- | --- | --- | --- | --- | --- | --- | --- | --- | --- |
| 1. | Family members should be given the option to be present when a loved one is being resuscitated. | | 1 | | 2 | | 3 | | 4 | 5 |
| 2. | Family members will panic if they witness a resuscitation effort. (reverse) | | 1 | | 2 | | 3 | | 4 | 5 |
| 3. | Family members will have difficulty adjusting to the long term emotional impact of watching a resuscitation effort. (reverse) | | 1 | | 2 | | 3 | | 4 | 5 |
| 4. | The resuscitation team may develop a close relationship with family members who witness the efforts, as compared to family members who do not witness the efforts. | | 1 | | 2 | | 3 | | 4 | 5 |
| 5. | I would be more anxious about doing things right if family members were present during a resuscitation effort. (deleted) (reverse) | | 1 | | 2 | | 3 | | 4 | 5 |
| 6. | If my loved one were being resuscitated, I would want to be present in the room. | | 1 | | 2 | | 3 | | 4 | 5 |
| 7. | Patients do not want family members present during a resuscitation attempt. (reverse) | | 1 | | 2 | | 3 | | 4 | 5 |
| 8. | The resuscitation team will try more extensive interventions if family members are present. (deleted) (reverse) | | 1 | | 2 | | 3 | | 4 | 5 |
| 9. | Family members who witness unsuccessful resuscitation efforts will have a better grieving process. | | 1 | | 2 | | 3 | | 4 | 5 |
| 10. | If my loved one were being resuscitated, I should be allowed to be present because I am a nurse. (deleted) | | 1 | | 2 | | 3 | | 4 | 5 |
| 11. | Family members will become disruptive if they witness resuscitation efforts. (reverse) | | 1 | | 2 | | 3 | | 4 | 5 |
| 12. | Family members who witness a resuscitation effort are more likely to sue. (reverse) | | 1 | | 2 | | 3 | | 4 | 5 |
| 13. | The resuscitation team will not function as well if family members are present in the room. (reverse) | | 1 | | 2 | | 3 | | 4 | 5 |
| 14. | Nurses with whom I work are not supportive of family presence during resuscitation efforts. (reverse) (deleted) | | 1 | | 2 | | 3 | | 4 | 5 |
| 15. | Family members on the unit where I work prefer to be present in the room during resuscitation efforts. | | 1 | | 2 | | 3 | | 4 | 5 |
| 16. | The presence of family members during resuscitation efforts is beneficial to patients. | | 1 | | 2 | | 3 | | 4 | 5 |
| **Please circle the number that best represents the extent to which you agree or disagree with the following statements:**  **The presence of family members during resuscitation efforts……..** | | Strongly Disagree | | Disagree | | Neutral | | Agree | | Strongly Agree |
| 17. | is beneficial to families. | 1 | | 2 | | 3 | | 4 | | 5 |
| 18. | is beneficial to nurses. | 1 | | 2 | | 3 | | 4 | | 5 |
| 19. | is beneficial to physicians. | 1 | | 2 | | 3 | | 4 | | 5 |
| 20. | should be a component of family-centered care. | 1 | | 2 | | 3 | | 4 | | 5 |
| 21. | will have a positive effect on patient ratings of satisfaction with hospital care. | 1 | | 2 | | 3 | | 4 | | 5 |
| 22. | will have a positive effect on family ratings of satisfaction with hospital care. | 1 | | 2 | | 3 | | 4 | | 5 |
| 23. | will have a positive effect on nurse ratings of satisfaction in providing optimal patient and family care. | 1 | | 2 | | 3 | | 4 | | 5 |
| 24. | will have a positive effect on physician ratings of satisfaction in providing optimal patient and family care. | 1 | | 2 | | 3 | | 4 | | 5 |
| 25. | is a right that all patients should have. | 1 | | 2 | | 3 | | 4 | | 5 |
| 26. | is a right that all family members should have. | 1 | | 2 | | 3 | | 4 | | 5 |
|  | | | | | | | | | | |
|  | **Please read each numbered item below and circle the number to indicate how confident you are that you could perform the listed behavior during a resuscitation effort with family members present.** | Not at all Confident | | Not Very Confident | | Somewhat Confident | | Quite Confident | | Very Confident |
| 27. | I could communicate about the resuscitation effort to family members who are present. | 1 | | 2 | | 3 | | 4 | | 5 |
| 28. | I could administer drug therapies during resuscitation efforts with family members present. | 1 | | 2 | | 3 | | 4 | | 5 |
| 29. | I could perform electrical therapies during resuscitation efforts with family members present. | 1 | | 2 | | 3 | | 4 | | 5 |
| 30. | I could deliver chest compressions during resuscitation efforts with family members present. | 1 | | 2 | | 3 | | 4 | | 5 |
| 31. | I could communicate effectively with other health team members during resuscitation efforts with family members present. | 1 | | 2 | | 3 | | 4 | | 5 |
| 32. | I could maintain dignity of the patient during resuscitation efforts with family members present. | 1 | | 2 | | 3 | | 4 | | 5 |
| 33. | I could identify family members who display appropriate coping behaviors to be present during resuscitation efforts. | 1 | | 2 | | 3 | | 4 | | 5 |
| 34. | I could prepare family members to enter the area of resuscitation of their family member. | 1 | | 2 | | 3 | | 4 | | 5 |
|  | **Please read each numbered item below and circle the number that indicates how confident you are that you could perform the listed behavior during a resuscitation effort with family members present.** | Not at all Confident | | Not Very Confident | | Somewhat Confident | | Quite Confident | | Very Confident |
| 35. | I could enlist support from attending physicians for family presence during resuscitation efforts. | 1 | | 2 | | 3 | | 4 | | 5 |
| 36. | I could escort family members into the room during resuscitation of their family member. | 1 | | 2 | | 3 | | 4 | | 5 |
| 37. | I could announce family member’s presence to resuscitation team during resuscitation efforts of their family member. | 1 | | 2 | | 3 | | 4 | | 5 |
| 38. | I could provide comfort measures to family members witnessing resuscitation efforts of their family member. | 1 | | 2 | | 3 | | 4 | | 5 |
| 39. | I could identify spiritual and emotional needs of family members witnessing resuscitation efforts of their family member. | 1 | | 2 | | 3 | | 4 | | 5 |
| 40. | I could encourage family members to talk to their family member during resuscitation efforts. | 1 | | 2 | | 3 | | 4 | | 5 |
| 41. | I could delegate tasks to other nurses in order to support family members during resuscitation efforts of their family member. | 1 | | 2 | | 3 | | 4 | | 5 |
| 42. | I could debrief family after resuscitation of their family member. | 1 | | 2 | | 3 | | 4 | | 5 |
| 43. | I could coordinate bereavement follow-up with family members after resuscitation efforts of their family member, if required. | 1 | | 2 | | 3 | | 4 | | 5 |

**Please select the answer that is true of you.**

44. If you were a patient who was being resuscitated, would you want your family members to be present in the room?

____Yes

____No

45. Have you ever been present in the room during the resuscitation of one of your family members?

____Yes

____No

46. How many times have you invited a family member to be present during a resuscitation attempt at BMH?

____Never

____Less than five times

____More than five times

47. On what unit were you working the last time that you invited a family member to be present during a resuscitation attempt?

____Emergency Department

____Critical Care Unit

____Non-Critical Care Inpatient Unit

____Other _________________________________________________

____Not Applicable

48. Who should make the decision about family presence during resuscitation efforts? Choose all that apply.

Patient (beforehand) ____Yes ____No

Nurse ____Yes ____No

Physician ____Yes ____No

Family ____Yes ____No

Other ______________________________________________________

49. Who is the BEST one to make the decision about family presence during resuscitation efforts? Choose one.

____Patient (beforehand)

____Family

____Nurse

____Physician

50. Should the decision about family presence be a part of an advanced directive authorized by the patient?

____Yes

____No

51. What type of unit do you work on most often?

____Emergency Department

____Critical Care Unit

____Non-Critical Care Inpatient Unit

____Outpatient Unit

____Other __________________________________________________

**Please select the option that best describes YOUR:**

**(Recall that you may omit any item that you wish)**

52. Current nursing role

____RN

____LPN

53. Years of experience in nursing

____Less than 1 year

____1 – 5 years

____6 – 10 years

____11 – 20 years

____More than 20 years

54. Highest nursing degree completed

____Licensed Practical Nurse Program

____Associate Degree in Nursing

____Baccalaureate Degree in Nursing

____Master’s Degree in Nursing

____Doctoral Degree in Nursing

55. Gender

____Male

____Female

56. Age

____18-24 years

____25-39 years

____40-55 years

____Over 56 years

57. Do you hold a specialty nursing certification?

____Yes (please list__________________________________________)

____No

58. Do you hold membership in a professional nursing organization?

____Yes

____No

59. Ethnicity

____African-American

____Asian

____Caucasian

____Hispanic

____Native American – Eskimo

____Pacific-Islander

____Other

60. What do you believe is your area of clinical expertise?_________________________________

61. The main reason I **would not** invite a family member into a code is:

62. The main reason I **would** invite a family member into a code is:

63. In the space below or on additional pages, please share with us any other opinions, stories or perspectives about family-witnessed resuscitation.

Thank you for your responses!

/rlm-renee twibell-fwnursesurvey-6-10-04
